# Methylglyoxal-Glyoxalase 1 Balance: The Root of Vascular Damage

**DOI:** 10.3390/ijms18010188

**Published:** 2017-01-18

**Authors:** Cecilia Nigro, Alessia Leone, Gregory Alexander Raciti, Michele Longo, Paola Mirra, Pietro Formisano, Francesco Beguinot, Claudia Miele

**Affiliations:** 1 Research Unit (URT) of the Institute of Experimental Endocrinology and Oncology “G. Salvatore”, National Council of Research, 80131 Naples, Italy; cecilia.nigro@alice.it (C.N.); aleleone86@libero.it (A.L.); gregoryraciti@gmail.com (G.A.R.); mi_longo@libero.it (M.L.); paolamirra.lib@libero.it (P.M.); fpietro@unina.it (P.F.); beguino@unina.it (F.B.); 2 Department of Translational Medical Sciences, University of Naples “Federico II”, 80131 Naples, Italy

**Keywords:** methylglyoxal, glyoxalase, vascular function, insulin-resistance

## Abstract

The highly reactive dicarbonyl methylglyoxal (MGO) is mainly formed as byproduct of glycolysis. Therefore, high blood glucose levels determine increased MGO accumulation. Nonetheless, MGO levels are also increased as consequence of the ineffective action of its main detoxification pathway, the glyoxalase system, of which glyoxalase 1 (Glo1) is the rate-limiting enzyme. Indeed, a physiological decrease of Glo1 transcription and activity occurs not only in chronic hyperglycaemia but also with ageing, during which MGO accumulation occurs. MGO and its advanced glycated end products (AGEs) are associated with age-related diseases including diabetes, vascular dysfunction and neurodegeneration. Endothelial dysfunction is the first step in the initiation, progression and clinical outcome of vascular complications, such as retinopathy, nephropathy, impaired wound healing and macroangiopathy. Because of these considerations, studies have been centered on understanding the molecular basis of endothelial dysfunction in diabetes, unveiling a central role of MGO-Glo1 imbalance in the onset of vascular complications. This review focuses on the current understanding of MGO accumulation and Glo1 activity in diabetes, and their contribution on the impairment of endothelial function leading to diabetes-associated vascular damage.

## 1. Introduction

Methylglyoxal (MGO) is a dicarbonyl aldehyde mainly formed as byproduct of glycolysis [1]. By its nature, MGO efficiently reacts with other molecules in the organism and causes cell and tissue dysfunction [2]. While cells use glucose as major source of energy and store most of their glucose in non-reactive molecules, as glycogen, a spontaneous and unavoidable leakage from the Embden-Meyerhof pathway occurs in mammalian cells in the form of MGO [1]. It has been estimated that about 0.1% of the glucotriose flux generates MGO by the non-enzymatic degradation of glyceraldehyde 3-phosphate (G3P) and dihydroxyacetone phosphate (DHAP) [3]. Thus, glycolysis contributes to the formation of MGO with a rate of about 125 µmol/kg cell mass per day under normoglycaemic conditions [4]. This situation may be further increased under conditions that lead to higher triosephosphate concentrations, like increased glucose metabolism in hyperglycaemia [5], impairment of the pentose pathway decreasing GA3P disposal, or increased anaerobic glycolysis occurring in hypoxia [6]. A minor amount of MGO is generated by acetone oxidation in the catabolism of ketone bodies [7], aminoacetone oxidation in the catabolism of threonine [8], lipid peroxidation and degradation of glycated proteins and monosaccharides [9,10].

At variance, the contribution of exogenous sources is still controversial. Extremely high levels of MGO were found in natural products, such as honey, and especially processed food and beverages like soft drinks, coffee, and dairy products [11,12]. Indeed, MGO levels in foodstuffs is strongly affected by heating [13], prolonged storage and fermentation, when microorganisms release MGO in alcoholic drinks and cheese [11].Unexpectedly, variable levels of MGO can be found even in drinking water that underwent treatments like ozonation and chlorination, which easily result in the formation of MGO from humic substances [14]. It should be further considered that polluted air also represents an exogenous source of MGO. In particular, smoking is a major source of indoor air contamination [15]. However, whether these exogenous sources of MGO are significant for plasma MGO levels has not been clarified yet. Several studies report that free MGO is rapidly degraded during digestion in the intestine and, thus, does not influence MGO level in vivo [16]. But there is also conflicting evidence indicating brain and serum MGO accumulation in rodents fed MGO-supplemented diets and suggesting that at least 10% of MGO-derived AGEs is absorbed and then accumulated in tissues like vessels, heart, liver, kidney and adipose tissue [17,18].

MGO levels in healthy humans have been estimated to be about 50–150 nM in the plasma and 1–4 µM in tissues [19]. When the accumulation of MGO exceeds these levels, dicarbonyl stress occurs as a consequence of the imbalance between the generation/exposure and MGO metabolism [20].

Under physiological conditions, >99% of MGO is detoxified by the glyoxalase system [21], with minor metabolism by aldoketo reductases (AKRs) and aldehyde dehydrogenases (ADHs) which convert MGO to hydroxyacetone and pyruvate, respectively [22]. The glyoxalase system consists of glyoxalase 1 (Glo1), glyoxalase 2 (Glo2) and a catalytic amount of glutathione (GSH). Acting as the rate-limiting enzyme, Glo1 catalyses the primary detoxification step by converting the spontaneously formed MGO-GSH hemithioacetal to the thioester *S*-d-lactoylglutathione [23]. Its activity is directly proportional to GSH concentration. Thus when cellular GSH concentration declines, as in oxidative stress, Glo1 activity is impaired. Glo2 catalyses the hydrolysis of *S*-d-lactoylglutathione to d-lactate and GSH [24]. With the exception of certain protozoa [25], both Glo1 and 2 are expressed in most eukaryotes and prokaryotes [26,27], and are localized in the cytoplasmic compartment [28].

The modulation of Glo1 activity is dependent on both regulation of gene expression and post-translational modifications. The Glo1 gene contains regulatory elements including ARE (antioxidant-response element) in exone 1 [29], and its expression is negatively regulated by HIF1α (hypoxia inducible factor 1α) binding to ARE under hypoxic conditions [30]. Indeed, hypoxia is an important physiological driver of dicarbonyl stress as it facilitates MGO formation by increasing metabolic flux through anaerobic glycolysis [6]. Hypoxia decreases Glo1 expression as well [3]. Furthermore, basal and inducible expression of Glo1 is subjected to stress-responsive control by nuclear factor erythroid 2-related factor 2 (Nrf2) through regulatory AREs [29]. Current evidence indicates that NF-κB constitutive activation in chronic hyperglycaemia, and related inflammation, mediates the impairment in Glo1 expression and activity [31]. It has been further demonstrated that NF-κB antagonizes the transcriptional activity of Nrf2 [32], suggesting that in a pro-inflammatory state, typical of diabetes and obesity, NF-κB activation impairs Glo1 expression by inhibiting Nrf2 action. Glo1 expression is also negatively regulated by the activation of the receptor of advanced glycated end products (RAGE) and, again, NF-κB has an important role in this pathway [33].

While genetic polymorphisms of Glo2 are extremely rare, different SNPs (single nucleotide polymorphisms) have been identified in the Glo1 gene and found to be associated with reduced enzyme activity [34], increased prevalence of diabetic neuropathy [35], and increased risk of cardiovascular complications [36]. Moreover, Glo1 is a hotspot for functional copy number variation (CNV) causing 2 to 4-fold increases in Glo1 expression in approximately 2% of the human population [37,38]. However, further studies are needed to understand whether these patients are protected from MGO accumulation and damage.

A physiological decrease of Glo1 activity and expression occurs with ageing, as demonstrated by Morcos et al. in the nematode *Caenorhabditis elegans*, where an inverse correlation was found between ageing and Glo1 expression [39]. This same effect has been confirmed in rodents [40,41,42]. Glo1 expression is therefore likely linked to healthy ageing [43].

**Figure 1:**
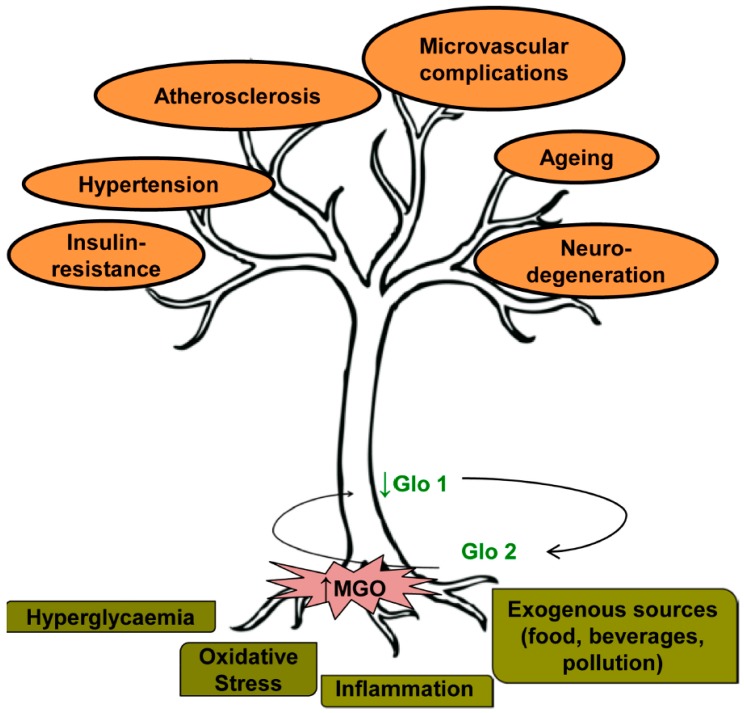
Sources of methylglyoxal (MGO) accumulation contributing to vascular dysfunction. Hyperglycaemia, oxidative stress, inflammation and exogenous sources of MGO contribute both to the increase of MGO levels and the decrease of glyoxalase 1 (Glo1) activity. MGO-Glo1 imbalance leads to vascular dysfunction contributing to endothelial insulin-resistance, hypertension, atherosclerosis, microvascular complications, ageing and neuro-degeneration.

Thus, abnormal cellular accumulation of the dicarbonyl metabolite MGO occurs upon exposure to high glucose concentrations, oxidative stress, inflammation, cell aging and senescence. It is associated with increased MGO-adduct content of proteins susceptible to dicarbonyl modification, collectively defined as dicarbonyl proteome [20], and with DNA instability and mutations [36,43]. An adequate balance between MGO levels and Glo1 activity is necessary to ensure detoxification of MGO from different sources and cell survival (Figure 1).

## 2. Effect of MGO Accumulation on Vascular Function

In the last decades, an increasing number of studies claiming a central role for MGO in vascular dysfunction have been published [44]. Endothelial dysfunction is one of the major factors in the development of cardiovascular disease. It has been recently reviewed how different noxious agents induce endothelial dysfunction, promoting an amplified endoplasmic reticulum stress response [45]. Vascular endothelium behaves as an autocrine and paracrine organ regulating vascular homeostasis [46]. This is ensured by the tight control of the vascular tone, cell–cell interaction, permeability and coagulation systems through the production of the extracellular matrix components, and soluble factors in response to various stimuli.

When this balance is impaired, vasoconstriction may occur, along with leukocyte adherence, platelet activation, mitogenesis, pro-oxidation, thrombosis and impaired coagulation, vascular inflammation, and atherosclerosis [47]. Among the bioactive molecules generated by the endothelium, nitric oxide (NO) plays a pivotal role in the maintenance of vascular homeostasis. NO controls vascular tone by maintaining basal and induced vascular smooth muscle relaxation and opposing the action of potent contraction factors, including Angiotensin II (Ang II) and Endothelin-1 (ET-1). In addition, NO inhibits platelet activation and leukocyte adhesion and rolling, as well as cytokine-induced expression of VCAM (vascular cell adhesion molecule) and MCP-1 (monocyte chemotactic protein-1), thus promoting an anti-inflammatory action [47].

MGO accumulation, perpetuated by Glo1 down-regulation, is linked to age-related diseases, such as diabetes, obesity, disorders of the central nervous system and cardiovascular disease, which share endothelial dysfunction as a common pathological denominator [47,48,49,50]. MGO reacts predominantly with arginine residues on proteins, with methylglyoxal hydroimidazolone (MG-H1) being the most prevalent MGO-derived AGE modification found in vivo, leading to structural change, inactivation and degradation of target proteins [51]. Thus, increased levels of MGO impair endothelial function in various way and in different districts in the organism. Several studies have shown an impairment of endothelium-dependent vasorelaxation by MGO, mostly mediated by oxidative stress. Indeed, acetylcholine-induced vasorelaxation is impaired in aortic tissue from rats by both high glucose and MGO. This effect is attenuated by the MGO scavengers aminoguanidine (AG) and *N*-acetyl-cisteine (NAC) [52], via the inhibition of NADPH oxidase [53], and is prevented by Glo1 over-expression [54]. A protective effect of anti-oxidants such as polyphenols has been reported to prevent MGO-dependent impairment of NO release, improving endothelium-mediated relaxation in mouse *corpora cavernosa* [55]. A proposed mechanism by which MGO increases reactive oxygen species (ROS), promoting the apoptotic process, involves the reduced transcription of the cytoprotective protein thioredoxin [56]. Moreover, accumulating evidence suggests that physiological angiogenesis is impaired by MGO through RAGE-mediated and autophagy-induced vascular endothelial growth factor receptor 2 (VEGFR2) degradation [57]. Our preliminary data indicate that Glo1 down-regulation in mouse aortic endothelial cells (MAECs) impairs the angiogenic process via a mechanism involving NFκB (unpublished data).

A study performed in Goto-Kakizaki diabetic rats demonstrates that endothelial dysfunction is worsened by MGO treatment, which increases oxidative stress, AGE formation and inflammation with a decline in NO bioavailability [58]. Moreover, MGO accumulation in arterial walls causes vascular contractile dysfunction in spontaneously hypertensive rats [59], and Dhar et al. have demonstrated that MGO treatment activates NFκB through RAGE, thereby increasing renin-angiotensin levels and blood pressure in Sprague-Dawley rats [60]. These findings provide further evidence that MGO is a causative factor in the pathogenesis of atherosclerosis and development of macrovascular diabetic complications. In humans, an association between MGO-derived AGEs and endothelial dysfunction markers has been found in individuals with type 1 diabetes [61,62]. In addition, reduced Glo1 activity in atherosclerotic lesions associates with increased HbA1c levels in non-diabetic patients [63].

As a pivotal mediator of endothelial-dependent release of NO and resulting smooth muscle relaxation, endothelial nitric oxide synthase (eNOS) represents a target of the harmful effect of MGO. Indeed, eNOS protein levels and its active phosphorylated form at the serine 1177 site are decreased in long-term MGO treatment of rat isolated mesenteric artery [53] and in thoracic aortic rings [64]. Also, eNOS uncoupling, found to be associated with O^−^_2_ generation and eNOS cofactor biopterin depletion [65], contributes to redox-sensitive leukocyte recruitment and microvascular leakage elicited by MGO [66]. In addition, age-related glycative and oxidative stress, resulting in endothelial dysfunction, is reduced in Glo1 transgenic rats [67].

Besides macrovascular function, MGO and MGO-derived AGEs also play a harmful effect on microvascular function, contributing to the onset of nephropathy and neuropathy. Indeed, regulation of the Glo1 enzyme has been proved to be important in prevention of early renal impairment in experimental diabetes [68], but also independently of hyperglycaemia in apoE^−/−^ mice [69]. This is also confirmed by the evidence that MGO accumulation in Wistar normal rats impairs several renal disease markers progressively observed in diabetic Goto-Kakizaki rats [70]. MGO induces blood–brain barrier damage by reducing the integrity and increasing the permeability of brain endothelial cells [71]. Recently, the generation of an occludin-MGO adduct, which leads to dysfunctional tight junctions and increased brain microvascular endothelial cell (BMEC) permeability, has been identified as a mechanism potentially involved in these abnormalities [72]. BMECs are crucial for brain vascular repair and maintenance. Recent evidence obtained from both in vitro [73,74] and in vivo [75] models of brain ischemia demonstrates that MGO induces BMEC injuries and exaggerates ischemia-reperfusion injury in diabetic rats.

Based on these findings, it became clearer that an effective reduction of MGO accumulation is crucial for preserving vascular function. Several attempts to alleviate dicarbonyl stress have been made in the last few decades, with MGO scavengers such as AG. But trials had to be terminated due to the lack of efficacy, safety concerns or undesired side effects [18]. A promising strategy is the development of Glo1 inducers through the activation and binding of Nrf2 to the Glo1 functional ARE. Up to now, a Glo1 inducer combination of *trans*-resveratrol and hesperetin (tRES-HESP) has been evaluated in a Phase 1 clinical trial and is now available for evaluation in Phase 2. In highly overweight subjects, tRES-HESP improves arterial dilation and decreases the vascular inflammation marker VCAM-1 [76]. Further efforts in the development of pharmacological intervention to prevent dicarbonyl stress are needed to provide new therapeutic options aimed at preventing vascular dysfunction in diabetes and other age-related diseases.

## 3. MGO-Induced Insulin-Resistance: A Link to Endothelial Dysfunction

Because of its highly reactive nature, MGO rapidly binds protein residues, with arginine and lysine representing the sites with the higher binding affinity [77]. This is particularly insidious as arginine and lysine are amino acid residues with a high probability of being located in the functional sites of proteins, including kinases involved in insulin signal transduction [3]. We and others have provided evidence about the role of MGO on insulin-resistance in major target tissues for insulin action.

MGO interferes with insulin signaling in L6 skeletal muscle and beta cells. In INS-1E beta cells it decreases insulin secretion through the inhibition of the insulin receptor substrate (IRS)/phosphatidylinositol 3-kinase (PI3K)/protein kinase B (Akt) pathway activation and independently from reactive oxygen species production. The inhibitory effect of MGO and the formation of AGE adducts on IRS are reversed by AG [78,79]. In addition, MGO administration in vivo inhibits insulin secretion from isolated beta cells, due to decreased PDX-1 levels, and results in higher levels of MGO-derived AGEs and insulin-resistance in muscle, liver, and adipose tissue [80,81,82]. Such effects are inhibited by the MGO scavenger Alagebrium, indicating the specificity of MGO effects. In support of animal data and studies in animal models, a very recent human study in healthy overweight individuals demonstrates that a diet with low AGE content decreases risk of type 2 diabetes by improving insulin sensitivity [83]. Besides the canonical target tissue of insulin action, MGO induces neuronal cell death in the central nervous system, and the impairment of insulin signaling was found in astrocytes exposed to MGO [84]. In both mice and humans, dietary MGO and AGEs were positively correlated with cognitive deficit, and inversely associated with the survival factor sirtuin-1 (SIRT1) levels and other markers of insulin sensitivity, suggesting MGO dependent SIRT1 down-regulation as a possible link between insulin-resistance and neurodegeneration [17].

Insulin also plays important haemodynamic actions. Insulin receptors were identified on endothelial cells of both large and small blood vessels by Jialal and colleagues in 1985 [85]. The hemodynamic effect of insulin was suggested by pivotal experiments reporting an insulin-mediated increase in blood flow into leg skeletal muscle during a hyperinsulinemic euglycaemic clamp [86]. Indeed, insulin action on endothelium is an integration of increased capillary recruitment and increased total blood flow. Both of these effects are reduced and delayed in insulin-resistance [87].

At the molecular level, insulin binding to its cognate receptor on endothelial cells activates IRS, PI3K and Akt, leading to the activation of eNOS [88]. eNOS catalyses the conversion of arginine to citrulline and NO. Its activity is regulated by phosphorylation at multiple sites; two of the best characterized sites are serine 1177 and threonine 495. Serine 1177 is rapidly phosphorylated by Akt in response to insulin, whereas threonine 495 is constitutively phosphorylated in endothelial cells and is thought to be a negative regulatory site [89,90]. Insulin is also able to cause rapid release of ET-1 via the mitogen-activated protein kinase (MAPK) cascade, which induces the activation of endothelin converting enzyme (ECE) in endothelial cells [50], followed by vasoconstriction and proliferation of vascular smooth muscle cells (VSMCs). Studies enabling the understanding of the causal role of insulin signal transduction in vasodilation were carried out in genetically modified animal models. Mice lacking IRS-1 feature hypertension and impaired endothelium-dependent vasorelaxation [91]. Consistently, a polymorphism at arginine 972 IRS-1 is associated with decreased eNOS expression in endothelial cells and plasma NO levels in human subjects [92]. Moreover, eNOS knock-out mice are hypertensive and have a 40% lower insulin-stimulated glucose uptake than control mice [93], indicating that insulin-mediated vasodilation and capillary recruitment are required for insulin delivery and action at target tissues.

**Figure 2:**
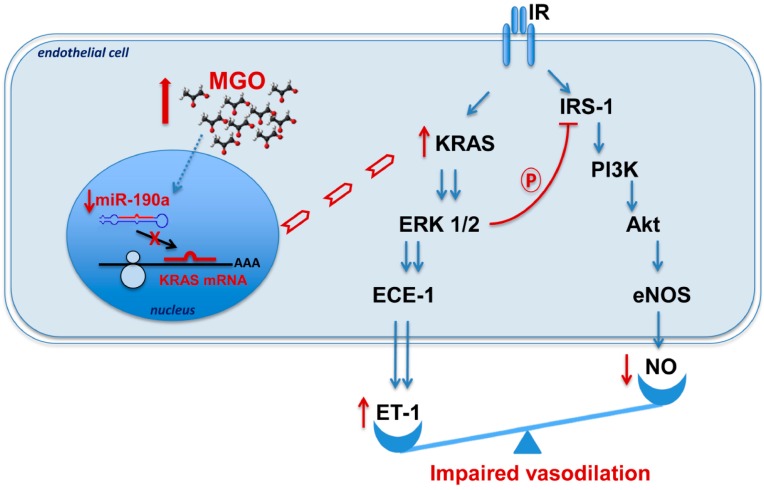
MGO-mediated endothelial insulin-resistance. MGO accumulation causes the reduction of miR-190a. Protein levels of miR-190a target KRAS increased, resulting in the hyperactivation of ERK 1/2. The latter phosphorylates IRS-1 (insulin receptor substrate 1) on serine 616 inhibiting its activation and the downstream pathway PI3K/Akt/eNOS. These effects result in the impairment of insulin stimulated NO production by endothelial cells, together with increased ET-1 release. Red ↑: increased molecule levels; red ↓: decreased molecule levels; blue ↓: protein activation; blue ↓↓: protein hyperactivation. KRAS (Kirsten rat sarcoma viral oncogene homolog).

Besides the above mentioned harmful effect of MGO on insulin-sensitivity of insulin target tissues, we have recently identified MGO as an endothelial insulin-resistance-inducing molecule (Figure 2) [94]. Increased MGO levels are responsible for the impairment of insulin action in mouse aortic endothelial cells (MAECs) exposed to exogenous MGO, or treated with the Glo1 inhibitor *S-p-bromobenzylglutathione cyclopentyl diester* (SpBrBzGSHCp2), and in vivo in healthy mice chronically treated with MGO. MGO exposure renders insulin unable to induce IRS-1 tyrosine phosphorylation, its association to p85, Akt activation and the subsequent eNOS phosphorylation on serine 1177, while the inhibitory phosphorylation of eNOS on threonine 497 is prevented by MGO accumulation. eNOS inactivation results in loss of the insulin-dependent increase in NO production. Conversely, ERK 1/2 activation is enhanced by MGO in both MAECs and aortic tissue from MGO-treated mice, accompanied by increased phosphorylation of IRS-1 on its inhibitory site serine 616. The rescue of IRS-1/Akt/eNOS pathway activation following the chemical inhibition of ERK 1/2 indicates that ERK 1/2 mediates, at least in part, the detrimental effect exerted by MGO on IRS-1/Akt pathway activation by phosphorylating IRS-1 at serine 616. Furthermore, ERK 1/2 hyperactivation does not result in increased MAEC proliferation, but rather in the increase of ET-1 production both in the absence and presence of insulin, suggesting that MGO alters endothelial insulin signaling and causes an imbalance between NO and ET-1 production, which facilitates vasoconstriction. Studies by Raoch et al. showed that NO regulates endothelin converting enzyme 1 ECE-1 expression [95]. It is therefore possible that the reduced NO levels also contribute to the activation of ECE-1 and the subsequent release of ET-1.

Emerging evidence suggests that MGO may alter gene expression through miRNA regulation [96,97], besides changes in DNA methylation [98] and histone modifications [99,100]. To answer the question of how MGO increases ERK 1/2 activation in MAECs, we have carried on studies which led to the recognition of miR-190a as an important modulator. The expression of this miRNA is down-regulated in both MAECs treated with MGO and aortic tissue from mice where Glo1 expression has been knocked down (Glo1-KD mice) [101]. The modulation of miR-190a inversely correlated with Kirsten rat sarcoma viral oncogene homolog (KRAS) levels and ERK 1/2 activation. Furthermore, pretreatment of MAECs with the inhibitor of class I and II histone deacetylase (HDAC), trichostatin A, was able to abolish the MGO effect on miR-190a expression, suggesting HDAC involvement. Consistent with previous findings proving the role of MGO in insulin-sensitivity and vascular function [53,55,78], the rescue of miR-190a levels we observed in MAECs upon the co-treatment with MGO and AG, revealed that MGO-modified proteins are critical in determining MGO-induced down-regulation of miR-190a.

Despite demonstration that MGO promotes insulin-resistance and hypertension by increasing oxidative stress [81], other investigators reported MGO-mediated insulin-resistance in skeletal muscle cells in the absence of ROS increases [79]. Whether oxidative stress may also play a part in MGO-mediated endothelial insulin-resistance remains to be further addressed.

## 4. Mouse Models of MGO Accumulation

To study the impact of MGO on vascular function, animal models that allow observation of the systemic implications of an imbalanced accumulation/detoxification ratio of MGO have been investigated. Important insights in this field have been generated by studies carried out in rodents that received MGO in drinking water [81], subcutaneous infusion with osmotic minipumps [80] or by chronic intraperitoneal infusion [94,102]. However, exogenous sources of MGO are only partially absorbed and not completely accumulated in tissues as free MGO, thus limiting the interpretation of these studies. Indeed, MGO rapidly reacts generating in situ adducts that, depending on the nature and half-life of the specific protein, can move in different ways to other districts in the organism or remain confined to the site of administration [22]. Experimental studies have often used MGO levels 10–20-fold higher than physiological concentrations. These conditions are similar to or exceed the upper limit of severe dicarbonyl stress in poorly controlled diabetes [103]. Moreover, MGO is physiologically formed intracellularly, as also indicated by the cellular MGO concentration exceeding that in plasma of human subjects by 100-fold [104]. Measuring tissue and plasma levels in experimental models is therefore necessary to ensure the patho-physiological relevance of the animal model.

A possibility to bypass these limitations is to modulate Glo1 activity and/or expression. Indeed, reduced activity of Glo1, inducible by treatment with the chemical Glo1 inhibitor SpBrBzGSHCp2 or by silencing Glo1 expression [105,106], mimics the physiological condition of endogenous accumulation of MGO as a consequence of MGO/Glo1 imbalance, occurring in both diabetes and ageing [107]. Conversely, Glo1 over-expression reduces hyperglycaemia-induced carbonyl stress [108], and has a protective effect on vascular dysfunction [68]. Chemical inhibition of Glo1 in apoE^−/−^ mice is able to increase endothelial inflammation and atherogenesis in the absence of hyperglycaemia, to a similar extent as in hyperglycaemic mice with diabetes [109]. Other studies using Glo1 genetically modified mice demonstrate its implication in microvascular complications. As reported by Giacco F. and colleagues, modulation of Glo1 in mice influences the risk of nephropathy development [106]. Indeed, in non-diabetic mice, knockdown of Glo1 increases both MGO modification of glomerular proteins and oxidative stress to diabetic levels, and causes alterations in kidney morphology which are indistinguishable from those caused by diabetes. Conversely, in streptozotocin (STZ)-induced diabetic mice, Glo1 over-expression prevents diabetes-induced increases in MGO modification of glomerular proteins, increased oxidative stress, and the development of diabetic kidney pathology, despite unchanged levels of diabetic hyperglycemia [106]. Recently, beneficial effects of Glo1 over-expression were also found in tubular cell survival, resulting in nephroprotective effects in renal ischemia-perfusion injury [110]. Another study of Bierhaus A. et al., demonstrated that both chemical Glo1 inhibition in wild-type mice and non-diabetic Glo1 knockdown mice develop signs of peripheral neuropathy, including thermal and mechanical hyperalgesia [105]. Strain dependent differences in Glo1 copy numbers also confirm that a lower expression of Glo1 promotes the development of diabetic neuropathy symptoms [111]. Very recent evidence obtained in Glo1 over-expressing mice has shown that MGO increases inflammation in diabetes, leading to endothelial cell loss and, thus, contributing to the development of diabetic cardiomyopathy [112]. Moreover, systemic Glo1 over-expression in rats prevents age-related impairment of endothelium-dependent vasorelaxation through modulation of eNOS phosphorylation, proving that blunting glycative stress prevents the long-term impact of endothelial dysfunction on vascular ageing [67]. In line with these findings, our preliminary data obtained in Glo1 knockdown mice indicated that Glo1 down-regulation is sufficient to increase blood pressure with ageing, likely as a consequence of impaired endothelium-dependent vasorelaxation. By contrast, previous studies in the same model did not reveal any differences in blood pressure compared to wild-type mice [106]. In this study, however, blood pressure was recorded in young mice where, even in presence of early signs of kidney damage, hypertension seemed to be not clinically evident yet.

## 5. Conclusions

It is undeniable that MGO accumulation has harmful effects on vascular function, by inducing insulin-resistance, hypertension, atherosclerosis, neurodegenerative disease and diabetic microvascular complications. A large body of literature supports the concept that maintaining the MGO/Glo1 balance is crucial to guarantee the containment of MGO levels under the toxic threshold. To this purpose, it is necessary to adopt a multi-task strategy. It is indeed not sufficient to minimize MGO intake. Also, improving glucose control to treat vascular dysfunction is likely hampered by metabolic memory. In this context, a promising contribution might be provided by modulating Glo1 activity. At the moment, it is necessary to use optimal experimental models to further uncover the pathways contributing to MGO accumulation and its effects, as well as its real impact on physiological systems, fostering the development of novel and effective pharmacological interventions to prevent vascular dysfunction.
